# The Relationship Between Ego Virtues and Burnout Among Cardiovascular Patients: A Cross-Sectional Study

**DOI:** 10.7759/cureus.100984

**Published:** 2026-01-07

**Authors:** Osama A Fakahani, Yazeed S Aljohani, Ammar Y Alharbi, Ali M Alasiry, Saad Albugami, Rania Zahid

**Affiliations:** 1 College of Medicine, King Saud Bin Abdulaziz University for Health Sciences, Jeddah, SAU; 2 Research Affairs, King Abdullah International Medical Research Center, Jeddah, SAU; 3 Cardiology, King Faisal Cardiac Center, Ministry of National Guard Health Affairs, Jeddah, SAU

**Keywords:** burnout, cardiovascular, ego virtues, psychosocial development, saudi arabia

## Abstract

Introduction: Cardiovascular diseases are a major health concern affecting both the physical and psychological well-being of patients. This study investigates the relationship between ego virtues and burnout among cardiovascular patients at King Abdulaziz Medical City, National Guard Health Affairs (NGHA), King Faisal Cardiac Center (KFCC), Jeddah, conducted between June and August 2022. Additionally, this study assesses the severity of burnout and explores potential gender-related differences in burnout and ego virtues within this patient population.

Methods: A cross-sectional study was conducted among 272 outpatients aged ≥20 at NGHA, KFCC, Jeddah. The study utilized the Oldenburg Burnout Inventory (OLBI) and the Psychosocial Inventory of Ego Strength (PIES). Pearson correlation coefficient, independent t-test, and descriptive statistics were used for statistical analysis.

Results: Among the participants, a moderate negative correlation was observed between ego virtues and burnout (r = -0.48, p < 0.001). Participants exhibited moderate levels of burnout, with a mean total burnout (BT) score of 31 (SD = 7). Male patients had higher total ego virtues (t = 2.503, p = 0.013) and lower BT scores (t = -2.836, p = 0.005) compared with female patients.

Conclusion: Our findings demonstrate a moderate level of burnout and an inverse correlation with ego virtues, with female participants exhibiting higher levels of burnout. These findings suggest that ego virtues may serve as protective factors against burnout among cardiovascular patients. However, considering the cross-sectional design of this study, further research is needed to determine a causal relationship.

## Introduction

Cardiovascular disease (CVD) is a broad term for any disorder affecting the structure or function of the heart or blood vessels, including coronary heart disease, cerebrovascular disease, and peripheral arterial disease [1]. CVDs are the leading cause of death worldwide, accounting for 31% of all deaths annually; the number of CVD-related deaths increased by 21% between 2007 and 2017 [2]. CVDs and related deaths are a major concern in Saudi Arabia, as a large number of Saudis suffer from this problem, with approximately 45% of all deaths [3,4]. Various psychosocial factors, including low socioeconomic status, lack of social support, stress, depression, hostility, and adverse childhood experiences, significantly influence CVDs [5-7]. A cohort study shows that burnout affects physical recovery and lower quality of life after a first acute coronary artery syndrome episode [8].

Burnout is an important variable associated with an increased risk of CVD [9]. It is defined as emotional and physical exhaustion resulting from overreaction to excessive stress in one’s environment [10]. A commonly used scale, the Maslach Burnout Inventory, measures burnout; however, its wording can be one-sided as the items in each subscale are framed in the same direction. For example, all items on exhaustion and cynicism are negatively phrased, whereas all personal efficacy items are positively phrased [11]. An alternative measure of burnout was developed by Demerouti et al. The Oldenburg Burnout Inventory (OLBI) is a scale used to measure academic and job burnout. It evaluates burnout severity based on statements regarding exhaustion and disengagement. It has more balanced wording as the items include both positive and negative phrasing [11].

Research by Rosenman et al. categorized people into two personality types. Type A is competitive, time-urgent, and aggressive. Type B is relaxed, patient, and easygoing. It showed that coronary artery disease is twice as likely to develop in a type A personality than in a type B personality [12].

Erik Erikson's psychosocial development theory outlines eight stages that unfold throughout life and are initiated by challenges or crises. Each stage has two opposing psychological tendencies: positive (syntonic) and negative (dystonic). Integrating them yields an ego virtue/strength that facilitates crisis resolution and aids in resolving subsequent stages, contributing to a stable foundation for a core belief system [13,14].

The first stage of Erikson's psychosocial development theory is Infancy (trust vs. mistrust). Between birth and 18 months, infants learn to trust the world around them. A successful resolution leads to the virtue of hope. Failure leads to the maldevelopment of withdrawal. Early childhood (autonomy vs. shame and doubt) comes next. Between ages 1.5 and 3, children develop a sense of autonomy and independence. The virtue of will is gained upon successful resolution. Failure leads to the maldevelopment of compulsion. The third stage is play age (initiative vs. guilt). Between ages three and five, children develop a sense of initiative and feel secure in their ability to undertake activities. A successful resolution leads to the virtue initiative. Failure leads to the maldevelopment of inhibition. The fourth stage is school age (industry vs. inferiority). Between ages 5 and 12, children begin to discover their talents and interests and to learn that they are competent. A successful resolution leads to the virtue of competence. Failure leads to the maldevelopment of inertia (passivity).

Adolescence (Identity vs. Identity Confusion) is the fifth stage. Between ages 13 and 20, adolescents become more independent and begin to discover their identities. They learn to establish their identity. A successful resolution leads to the virtue of fidelity. Failure leads to the maldevelopment repudiation. The sixth stage is Young Adulthood (Intimacy vs. Isolation). Between ages 20 and 40, young adults seek love and companionship through romantic relationships and friendships. They develop long-term relationships and reciprocal commitments. A successful resolution leads to the virtue of love. Failure leads to the maldevelopment of distantiation. Adulthood (Generativity vs. Stagnation/Self-Absorption) is the seventh stage. Between the ages of 40 and 65, individuals ask whether they are contributing to society and whether they can make their lives count. A sense of generativity develops when individuals provide for their children, are involved in community activities, or are productive at work. A successful resolution leads to the virtue care. Failure leads to the maldevelopment of rejectivity. The last stage is old Age (Integrity vs. Despair). From the age of 65 years, individuals reflect on their lives. They learn to accept their lives and feel content with their contributions to society, thereby developing integrity. A successful resolution leads to the virtue of wisdom. Failure leads to the maldevelopment of disdain [13-15].

A 2021 study conducted in Saudi Arabia found a positive association between individuals' positive thinking and their ego virtues [16]. Markstrom et al. developed the Psychosocial Inventory of Ego Strengths (PIES) to assess the components of Erikson’s psychosocial theory [17].

The relationship between psychosocial development and burnout remains understudied, especially among populations where burnout has been recognized as a risk factor, such as patients diagnosed with CVD.

Our research aims to assess the relationship between ego virtues and burnout, evaluate the severity of burnout, and determine whether there are significant gender-based differences in burnout and ego virtues among cardiovascular patients in National Guard Health Affairs (NGHA), King Faisal Cardiac Center (KFCC), Jeddah, Saudi Arabia, between June and August 2022.

## Materials and methods

The study used a cross-sectional design and was conducted at King Abdulaziz Medical City, NGHA, KFCC, Jeddah, Saudi Arabia, between June and August 2022. Outpatients with CVD, both male and female patients aged ≥20 years, regardless of comorbidities, were included. There was no additional exclusion criterion.

The minimum sample size is 249 subjects, calculated using Raosoft (Raosoft, Inc., Seattle, WA) [18] for a population of approximately 700, with a 5% margin of error and a 95% confidence level. As no previous studies were conducted, we used 50% for the response distribution. This sample size is large enough to avoid sampling bias.

A nonprobability convenience sampling technique was used. Data collection was conducted through a questionnaire-based survey with three sections. The first section gathered sociodemographic information, including age, gender, marital status, education level, occupation, and the onset of heart condition in years (see Appendix 1).

The second section included the OLBI scale, which contains 16 items (B1-B16) divided into two dimensions. The first dimension, exhaustion (BD1), includes items B2, B4, B5, B8, B10, B12, B14, and B16 (eight items). The second dimension, disengagement (BD2), includes items B1, B3, B6, B7, B9, B11, B13, and B15 (eight items). Items B1, B5, B7, B10, B13, B14, B15, and B16 are scored as strongly agree = 1 to strongly disagree = 4. Items B2, B3, B4, B6, B8, B9, B11, and B12 the scoring is reversed, with strongly agree = 4 to strongly disagree = 1. The score for each dimension is calculated by summing the scores of the positive pole and negative pole items, resulting in a score ranging from 8 to 32 per dimension. The total burnout (BT) is calculated by summing the scores of the two dimensions (BD1 + BD2), giving a score ranging from 16 to 64, with higher scores indicating higher levels of burnout. Classification cutoffs reported by Leclercq et al. were used: BT scores: low <30, moderate 30-45, high >45; BD1: low <15, moderate 15-23, high >23; BD2: low <14, moderate 14-23, high >23 [19].

To ensure accuracy and equivalence in the translation of the OLBI manual, a back-translation method was employed, involving discussions and revisions among the research team and an independent linguistics expert in both Arabic and English. Approval was granted after consensus was achieved (see Appendix 2).

The third section included the PIES developed by Markstrom et al. [17], using the Arabic version translated and validated by Alghamdi [20]. The original 32-item PIES includes eight subscales, each representing a stage in Erikson’s theory. Each subscale has two themes, a positive and a negative pole, each with two items, for a total of four items per subscale. The research group supervisor consulted with Alghamdi to reduce the scale to 16 items (E1-E16), with two items per subscale: Hope, Will, Purpose, Competency, Fidelity, Love, Care, and Wisdom. Each subscale has two items, one for each pole. Items E2, E3, E5, E8, E9, E13, E15, and E16 are scored as strongly agree = 5 to strongly disagree = 1. Items E1, E4, E6, E7, E10, E11, E12, and E14 are scored in reverse, with strongly agree = 1 to strongly disagree = 5. Scores for each dimension are calculated by summing the positive pole and negative pole question scores, yielding a score between 2 and 10. The total ego strength (ET) is calculated by summing the scores of the eight dimensions, yielding a score between 16 and 80, with higher scores indicating greater psychosocial development (see Appendix 3).

An informed consent form was prepared, and consent was obtained verbally from all participants. Data were kept confidential. The study received approval from the Institutional Review Board at King Abdullah International Medical Research Center (IRB approval no.: IRB/0738/22).

Data were entered into Microsoft Excel (Microsoft Corporation, Redmond, WA) for data checking, proofreading, and cleaning. Numerical variables included total burnout (BT), ET, age, and the onset of heart condition in years. Categorical variables included gender, marital status, education level, and occupation.

Data analysis

Data were analyzed using Statistical Package for the Social Sciences version 25 (IBM Corp., Armonk, NY). Descriptive statistics were used to calculate the mean and SD for both the burnout and ego virtues samples. The Pearson correlation coefficient was used to assess the relationship between burnout and ego virtues. The independent sample t-test was used to compare mean values between male and female participants. Statistical significance was set at p < 0.05.

## Results

A total of 272 cardiovascular outpatients were recruited. Most were older than 60 years (43.4%), male (80.1%), married (82.7%), and retired (47.1%). One-quarter were high school graduates (25%). Most participants developed a heart condition in the past zero to one years (29.4%) and in the past more than six years (27.2%). Demographic data are presented in Table 1.

**Table 1:** Demographic variables

Variable	Category	Frequency	Percentage (%)
Age	20-39 years	46	16.9
	40-59 years	108	39.7
	+60 years	118	43.4
Gender	Male	218	80.1
	Female	54	19.9
Marital status	Single	33	12.1
	Married	225	82.7
	Divorced	5	1.8
	Widow	9	3.3
Education level	Postgraduate	12	4.4
	Bachelor’s degree	58	21.3
	Diploma	19	7
	High-school graduate	68	25
	Intermediate-school graduate	41	15.1
	Elementary-school graduate	37	13.6
	Illiterate	22	8.1
	No formal education (not illiterate)	15	5.5
Occupation	Government sector	71	26.1
	Private sector	19	7
	Freelancer	10	3.7
	Retired	128	47.1
	Housewife	24	8.8
Onset of heart condition in years	0-1 years	80	29.4
	2-3 years	65	23.9
	4-5 years	53	19.5
	+6 years	74	27.2

Table 2 presents descriptive statistics for the OLBI. For the exhaustion (BD1) and disengagement (BD2) dimensions, mean values were 15.25 (SD = 3.77) and 15.44 (SD = 3.93), respectively, indicating a moderate OLBI score in both dimensions. The mean value for BT was 31 (SD = 7), indicating a moderate total OLBI score. Reliability analysis was conducted on the 16-item OLBI, with a Cronbach’s alpha of 0.642.

**Table 2:** Descriptive statistics for OLBI OLBI: The Oldenburg Burnout Inventory; BD1: burnout dimension 1 (exhaustion); BD2: burnout dimension 2 (disengagement); BT: total burnout

Parameter	n	Mean	Standard deviation	Score
BD1	272	15.25	3.77	Moderate
BD2	272	15.44	3.93	Moderate
BT	272	31	7	Moderate

Descriptive statistics for the PIES are presented in Table 3. The mean ET score is 65 (SD = 7), with higher scores indicating greater psychosocial development. Reliability analysis was conducted on the 16-item PIES with a Cronbach’s alpha of 0.638.

**Table 3:** Descriptive statistics for PIES PIES: Psychosocial Inventory of Ego Strengths

Parameter	n	Mean	Standard deviation
Hope	272	8.33	1.74
Will	272	7.66	1.6
Purpose	272	8.9	1.5
Competency	272	7.1	1.9
Fidelity	272	9.1	1.3
Love	272	8.4	1.8
Care	272	8.3	1.7
Wisdom	272	7.1	2.2
Total ego strength	272	65	7

An independent samples t-test was performed to compare burnout between male and female participants. Female participants had significantly higher BT than male participants, t(270) = −2.836, p = 0.005. They also had significantly higher exhaustion (BD1), t(270) = -1.976, p = 0.049, as well as disengagement (BD2), t(270) = -2.996, p = 0.003, compared to male participants. Both male and female participants had moderate scores on BD1, BD2, and BT, as shown in Table 4.

**Table 4:** Comparison of burnout between males and females BD1: burnout dimension 1 (exhaustion); BD2: burnout dimension 2 (disengagement); BT: total burnout Significant at p < 0.05

Parameter	Gender	n	Mean	Standard deviation	Score	Independent t-test value	p value
BD1	Male	218	15.0	3.86	Moderate	-1.976	0.049
	Female	54	16.1	3.25	Moderate		
BD2	Male	218	15.1	3.91	Moderate	-2.996	0.003
	Female	54	16.9	3.75	Moderate		
BT	Male	218	30.1	6.85	Moderate	-2.836	0.005
	Female	54	33.0	6.09	Moderate		

Using an independent samples t-test to compare ego virtues between male and female participants, male participants scored significantly higher on the will dimension (ED2), t(270) = 2.08, p = 0.039, the love dimension (ED6), t(270) = 2.02, p = 0.047, the wisdom dimension (ED8), t(270) = 2.12, p = 0.035, and ET, t(270) = 2.50, p = 0.013. There were no significant differences between them on the other dimensions, as shown in Table 5.

**Table 5:** Comparison of ego virtues between male and female patients Significant at p < 0.05

Parameter	Gender	n	Mean	Standard deviation	Independent t-test value	p value
Hope	Male	218	8.4	1.74	1.585	0.114
	Female	54	8	1.70		
Will	Male	218	7.8	1.54	2.076	0.039
	Female	54	7.2	1.80		
Purpose	Male	218	9	1.53	1.097	0.273
	Female	54	8.7	1.53		
Competency	Male	218	7.1	1.89	-0.214	0.831
	Female	54	7.2	1.86		
Fidelity	Male	218	9.1	1.39	-0.097	0.923
	Female	54	9.1	1.14		
Love	Male	218	8.5	1.71	2.018	0.047
	Female	54	7.9	2.13		
Care	Male	218	8.4	1.66	1.442	0.150
	Female	54	8	1.92		
Wisdom	Male	218	7.3	2.26	2.120	0.035
	Female	54	6.6	1.96		
Total ego strength	Male	218	65.6	7.45	2.503	0.013
	Female	54	62.8	7.12		

Pearson correlation coefficients showed a moderate negative correlation between ET and burnout measures: ET with BT, r(270) = -0.489, p < 0.001; ET with BD1, r(270) = -0.432, p < 0.001; ET with BD2, r(270) = -0.430, p < 0.001, as shown in Table 6. These results indicate that higher ego strength scores were associated with lower BT, exhaustion, and disengagement.

**Table 6:** Pearson correlation coefficients between ego strength and burnout ET: total ego strength; BT: total burnout; BD1: burnout dimension 1 (exhaustion); BD2: burnout dimension 2 (disengagement) ^*^Significant at p < 0.01 (two-tailed)

Parameter	BT	BD1	BD2
ET	-0.489^*^	-0.432^*^	-0.430^*^

## Discussion

CVDs are a major health concern, especially in the Kingdom of Saudi Arabia (KSA), contributing to 201,300 Saudis with a cardiovascular condition, including 149,600 with ischemic heart disease and 51,700 with cerebrovascular disease, with 45% of all deaths [21]. The most prevalent modifiable risk factor in the KSA is dyslipidemia, affecting 35% of the population. This is significantly higher than that in other countries. For example, in the United States, approximately 21% of adults have dyslipidemia [22].

Several studies have reported high burnout rates across various medical specialties and healthcare professions. A meta-analysis of 215,787 participants found that 39% of the public health workforce globally reported burnout [23]. One study of nurses at Yanbu General Hospital reported that 67.5% experienced high burnout, with 50.6% experiencing intense emotional exhaustion, 29.3% experiencing high depersonalization, and 30.5% experiencing low personal accomplishment [24]. A high burnout rate has also been reported among healthcare workers in the Eastern Province of Saudi Arabia, with 67% experiencing exhaustion and 60% experiencing cynicism [25]. A study in Jeddah highlighted a significant prevalence of burnout among nurses [26]. Furthermore, 81.22% of resident physicians registered high scores on at least one burnout subscale [27]. Another study observed that emergency and surgery residents experienced burnout rates across subscales similar to those seen in other Saudi specialties [28].

Research has shown that burnout can significantly increase cardiovascular risk. A large-scale study of over 11,000 participants revealed a 45% increased risk of atrial fibrillation in those in the highest quartile of vital exhaustion. This remained significant at 20% after adjusting for comorbidities [29]. A meta-analysis of 26,916 participants revealed that burnout increased CVD risk by 21% in adjusted models and by 27% in crude models. Furthermore, it also showed that burnout contributes to an increase in the risk of prehypertension by 85% and a 10% increased risk of CVD-related hospitalization [30]. A multicohort study of 85,494 participants found that in comparison to those who worked a standard 35-40 hours a week, individuals working ≥55 hours a week had an approximately 40% increased risk of atrial fibrillation, with a dose-response hazard ratios of 1.02, 1.17, and 1.42 for 41-48, 49-54, and ≥55 weekly working hours, respectively [31]. In Chile, work-related burnout was linked to a 59% higher chance of comorbid systolic/diastolic hypertension. Individuals experiencing both personal and work-related burnout had a mean diastolic blood pressure increase of 1.66 mmHg and double odds of developing isolated diastolic hypertension [32].

Further research on related concepts included a study with diabetic patients, which found no difference in ego virtues compared to nondiabetics [33]. Another study on senior students established a positive link between ego virtues and stress management [34].

Our findings indicate a moderate, negative, and statistically significant relationship between the total score of ego strength and the total score of burnout and its dimensions. A study conducted in India among 729 females reported a significant negative relationship between ego strength and burnout, with a correlation coefficient of -0.336. It also showed a significant negative correlation between ego strength and other specific burnout components, including emotional exhaustion, depersonalization, and reduced personal accomplishment [35]. This suggests that individuals with higher ego strength are less likely to experience burnout, whereas those with lower ego strength may be more susceptible. This supports our findings of the negative correlation between ego virtues and burnout.

Another study conducted in India, among 50 female college teachers, found a significant negative correlation between emotional exhaustion and ego strength, and between depersonalization and ego strength. However, there was a statistically insignificant negative correlation coefficient of -0.265 between ego strength and burnout [36]. Despite not showing a significant negative correlation between ego strength and burnout, this study showed a significant negative correlation between ego strength and emotional exhaustion.

Our sample showed a moderate level of burnout overall among cardiovascular outpatients. In addition, the data reveal that male participants have significantly higher scores than female participants on the will, love, and wisdom dimensions, as well as ET. On the other hand, female participants scored significantly higher than their male counterparts in all burnout dimensions, exhaustion (BD1), disengagement (BD2), and BT scores. According to the Medscape National Physician Burnout, depression and suicide report 2019, a high percentage reported feeling burnt-out, as in our study, female participants reported more burnout than male participants [37]. Studies show gender differences in burnout. In a study of dental postgraduates, the prevalence of job burnout was 4.7% higher among female participants than among male participants [38]. A cross-sectional study on 136 patients with burnout showed female participants having higher levels of emotional exhaustion and reduced vitality and vigilance compared to male participants [39]. A meta-analysis of 183 studies found that women score higher on emotional exhaustion, while men score higher on depersonalization [40]. A study on university professors showed female participants had higher levels of perceived stress, emotional exhaustion, and neuroticism compared with male counterparts [41].

However, some studies found mixed or nonsignificant results. A survey of US nurse leaders during the COVID-19 pandemic found that female nurses experienced higher personal burnout, while male nurses experienced higher client-related burnout [42]. A study of endocrinologists in China found no significant gender differences in the overall prevalence of burnout [43]. On the other hand, ego virtues may manifest differently across genders, with each gender potentially showing strengths in different areas. A study found that women, on average, scored higher on the 3-Dimensional Wisdom Scale. Women also scored higher on compassion-related domains and self-reflection, while men scored higher on cognitive-related domains and emotion regulation [44]. A study of adolescents found that boys scored higher on projection and aggression-outward defenses, whereas girls scored higher on turning against the self [45]. Additionally, a study comparing middle-aged and older adults found that ego virtues were more pronounced in older adults for both genders, indicating greater development with age [46].

The inverse relationship between ego virtues and burnout suggests a potential benefit in incorporating psychosocial assessment into cardiovascular care. The potential role of ego virtues as a protective factor against burnout is also important, as individuals with stronger psychosocial resilience are better able to cope with stressors and complex environments and, therefore, are less likely to have burnout. This insight underscores the potential importance of integrating psychosocial resilience-building measures into health care practice, especially in individuals who are susceptible to burnout, including patients with health conditions like CVD.

Strengths and limitations

The main strengths of this cross-sectional study are its cost-effectiveness, relative quickness to conduct, and being an efficient choice for determining the prevalence rate, which helped us to study the association between ego viruses and burnout in cardiac patients. The subjects are neither deliberately exposed nor treated; thus, there are seldom ethical difficulties. The limitations of the study include the inability to assess incidence and the cross-sectional design, which limits our ability to infer causality. It may not account for changes in population over time, leading to misleading results when there are seasonal or temporal effects. Still, it generates a hypothesis for further investigations or in-depth analysis through other studies. The study was conducted within a single department at the KFCC in Jeddah and involved a relatively small number of participants. Consequently, the findings may not be generalizable to broader populations or other healthcare settings. We needed to select a sample of subjects from a large and heterogeneous study population. Thus, it was susceptible to sampling bias. Therefore, further longitudinal research is essential to confirm these associations and to understand better the mechanisms underlying the relationship between ego virtues and burnout in this patient population. Future studies should aim to include a more diverse sample and consider these variables to enhance the generalizability and depth of the findings.

## Conclusions

This study examines the relationship between burnout and ego virtues among cardiovascular patients at KFCC. The findings indicate that the sample experienced moderate burnout, as reflected in overall scores and across the specific dimensions assessed. Notably, an inverse relationship was observed between ego virtues and burnout levels, suggesting that lower ego virtues are associated with higher burnout. Additionally, gender differences were evident, with female patients demonstrating higher burnout levels and lower total ego virtues compared to their male counterparts.

These findings highlight the potential significance of ego virtues as a protective factor against burnout in patients with CVD. Consequently, understanding the relationship between ego virtues and burnout, as well as examining patients’ psychosocial status, may inform the development of more effective strategies. Such strategies could include integrating psychosocial resilience-building measures into healthcare practices and enhancing burnout management, thereby reducing the overall burden of CVD. In light of the acknowledged limitations of this study, future research should target a more diverse population, including individuals with various health concerns, and use different study designs.

## Appendices

Appendix 1

**Table 7:** Demographics variables

Questions	
Age/العمر	
Gender/الجنس	Male/ذكر ☐ Female/أنثى ☐
Marital status/الحالة الزوجية	Single/أعزب ☐ Married/متزوج ☐
Educational level/المستوى التعليمي	Postgraduate/الدراسات العليا ☐ Bachelor’s degree/بكالوريوس ☐ Diploma/شهادة دبلوم ☐ High-school graduate/شهادة الثانوي ☐ Intermediate-school graduate/شهادة المتوسط ☐ Elementary-school graduate/شهادة الابتدائي ☐ Illiterate/أمي ☐ No formal education/لا يوجد ☐
Occupation/المهنة	Government sector/قطاع حكومي ☐ Private sector/قطاع خاص ☐ Freelancer/أعمال حرة ☐ Retired/متقاعد ☐ Housewife/ربة منزل ☐
When were you diagnosed with a heart condition?/متى ظهرت مشكلة القلب؟	D/يوم: ____ M/شهر: ____Y/سنة: ____

Appendix 2

Oldenburg Burnout Inventory Manual Questionnaire and Response Rate

**Table 8:** Oldenburg Burnout Inventory Manual B1: burnout question 1; B2: burnout question 2; B3: burnout question 3; B4: burnout question 4; B5: burnout question 5; B6: burnout question 6; B7: burnout question 7; B8: burnout question 8; B9: burnout question 9; B10: burnout question 10; B11: burnout question 11; B12: burnout question 12; B13: burnout question 13; B14: burnout question 14; B15: burnout question 15; B16: burnout question 16

Questions	Strongly disagree, n (%)	Disagree, n (%)	Agree, n (%)	Strongly agree, n (%)
B1. I always find new interesting aspects in my work أجد دائمًا جوانب جديدة ومثيرة للاهتمام في عملي	21 (7.7)	23 (8.5)	82 (30.1)	146 (53.7)
B2. There are days when I feel tiered before I arrive at work هنالك أيام أشعر فيها بالتعب قبل وصولي إلى العمل	78 (28.7)	75 (27.6)	80 (29.4)	39 (14.3)
B3. It happens more and more often that I talk about my work in a negative way يحدث في كثير من الأحيان أنني أتحدث عن عملي بطريقة سلبية	165 (60.7)	59 (21.7)	30 (11)	18 (6.6)
B4. After work, I tend to need more time than in the past to relax and feel better بعد العمل، أميل للحاجة إلى مزيد من الوقت أكثر مما كنت في الماضي للاسترخاء والشعور بالتحسن	60 (22.1)	50 (18.4)	86 (31.6)	76 (27.9)
B5. I can tolerate the pressure of my work very well أستطيع أن أتحمل ضغط عملي جيداً	5 (1.8)	19 (7)	68 (25)	180 (66.2)
B6. Lately, I tend to think less at work and do my job almost mechanically في الآونة الأخيرة، أميل إلى التفكير بشكل أقل في العمل وأقوم بعملي بشكل ميكانيكي تقريباً	87 (32)	48 (17.6)	59 (21.7)	78 (28.7)
B7. I find my work to be positive challenge أجد أن عملي يمثل تحديا إيجابياً	9 (3.3)	18 (6.6)	64 (23.5)	181 (66.5)
B8. During my work, I often feel emotionally drained أثناء عملي، غالبا ما أشعر بالاستنزاف العاطفي	144 (52.9)	68 (25)	44 (16.2)	16 (5.9)
B9. Overtime, one can become dis-connected from this type of work بمرور الوقت، يمكن أن يصبح المرء غير متصل بهذا النوع من العمل	105 (38.6)	57 (21)	55 (20.2)	55 (20.2)
B10. After working, I have enough energy for my leisure activities بعد العمل، لدي طاقة كافية لأنشطة أوقات الفراغ	24 (8.8)	51 (18.8)	80 (29.4)	117 (43)
B11. Sometimes I feel sickened by my work task أشعر أحياناً بالغثيان بسبب مهام عملي	173 (63.6)	53 (19.5)	35 (12.9)	11 (4)
B12. After my work I usually feel worn out and weary بعد عملي أشعر عادةً بالإرهاق والتعب	61 (22.4)	79 (29)	83 (30.5)	49 (18)

Appendix 3

Psychosocial Inventory of Ego Strength Questionnaire and Response Rate

**Table 9:** Psychosocial Inventory of Ego Strength E1: Erikson’s question 1; E2: Erikson’s question 2; E3: Erikson’s question 3; E4: Erikson’s question 4; E5: Erikson’s question 5; E6: Erikson’s question 6; E7: Erikson’s question 7; E8: Erikson’s question 8; E9: Erikson’s question 9; E10: Erikson’s question 10; E11: Erikson’s question 11; E12: Erikson’s question 12; E13: Erikson’s question 13; E14: Erikson’s question 14; E15: Erikson’s question 15; E16: Erikson’s question 16

Questions	Strongly disagree, n (%)	Disagree, n (%)	Natural, n (%)	Agree, n (%)	Strongly agree, n (%)
E1. I find I can easily be distracted even when I really need to finish a task يمكن تشويشي بسهولة، حتى لو كنت في حاجة لإنهاء المهمة التي بين يدي	92 (33.8)	55 (20.2)	65 (23.9)	46 (16.9)	14 (5.1)
E2. No matter how bad things get, I am confident they will get better مهما ساءت الأمور، فإني على ثقة من أنها ستكون أفضل	0 (0)	8 (2.9)	10 (7.4)	65 (23.9)	178 (65.4)
E3. When I see someone with a need, I help in whatever way I am able عندما أرى شخصاً في حاجة للمساعدة، فإنني أساعده بأي طريقة ممكنة	0 (0)	6 (2.2)	15 (5.5)	31 (11.4)	220 (80.9)
E4. I really don't know what strength or skills I have to offer society لا أعرف نقاط القوة والمهارات التي يجب أن أقدمها للمجتمع	86 (31.6)	54 (19.9)	75 (27.6)	37 (13.6)	20 (7.4)
E5. I am involved in a variety of activities that allow me to use my skills and abilities أشارك في العديد من الأنشطة التي تمكني من استخدام قدراتي ومهاراتي	36 (13.2)	16 (5.9)	69 (25.4)	59 (21.7)	92 (33.8)
E6. I don't think I have an intimate relationship from outside of my family لا أرتبط بعلاقات متينة مع أفراد من خارج نطاق أسرتي	152 (55.9)	39 (14.3)	29 (10.7)	16 (5.9)	36 (13.2)
E7. I really don't know what I want out of life أنا في الحقيقة لا أعرف ماذا أريد في هذه الحياة	218 (80.1)	27 (9.9)	12 (4.4)	7 (2.6)	8 (2.9)
E8. When I make a commitment to something I stick with it عندما التزم بشيء فإنني أحافظ على التزامي وأتقيد به	0 (0)	4 (1.5)	23 (8.5)	39 (14.3)	206 (75.7)
E9. In many ways, I have control over my future لدي القدرة على ضبط مستقبلي والتحكم فيه بطرق مختلفة	7 (2.6)	13 (4.8)	52 (19.1)	86 (31.6)	114 (41.9)
E10. Beyond my closest friends and family, I'm not concerned about the need of other people فيما عدا أسرتي وأصدقائي الحميمين، فإنني لا اهتم كثيرا بحاجات الآخرين	114 (41.9)	46 (16.9)	43 (15.8)	25 (9.2)	44 (16.2)
E11. I'm not sure what I believe in لست متأكد مما أعتقده في هذه الحياة	207 (76.1)	27 (9.9)	15 (5.5)	10 (3.7)	13 (4.8)
E12. When I reflect on the past, I feel sadness and regret عندما أفكر في الماضي فإنني اشعر بالحزن والندم	89 (32.7)	40 (14.7)	55 (20.2)	35 (12.9)	53 (19.5)
E13. I'm not afraid of what the future has in store for me لا يقلقني ما يخبئ المستقبل لي	34 (12.5)	26 (9.6)	34 (12.5)	28 (10.3)	150 (55.1)
E14. When something doesn't work out the way I hoped, it makes me feel like just quitting everything عندما لا تعمل الأشياء بالطريقة التي أتمناها، فإن ذلك يشعرني بالرغبة في إيقاف كل شيء	130 (47.8)	44 (16.2)	46 (16.9)	24 (8.8)	28 (10.3)
E15. My friends and I believe we can disagree on things and still be friends نؤمن أنا وأصدقائي بقدرتنا على الاحتفاظ بصداقتنا رغم اختلافنا حول بعض القضايا	5 (1.8)	6 (2.2)	35 (12.9)	45 (16.5)	181 (66.5)
E16. Even though I'm sometimes afraid of failing, if there something I want to do, I try to do it بالرغم من خوفي من الفشل، فإنني أحاول القيام بما أرغب القيام به	12 (4.4)	10 (3.7)	31 (11.4)	43 (15.8)	176 (64.7)

## References

[1] Thiriet M (2018). Cardiovascular disease: an introduction. Biomathematical and Biomechanical Modeling of the Circulatory and Ventilatory Systems.

[2] Emamian MH, Hashemi H, Fotouhi A (2020). Predicted 10-year risk of cardiovascular disease in the Islamic Republic of Iran and the body mass index paradox. East Mediterr Health J.

[3] Alqahtani BA, Alenazi AM (2024). A national perspective on cardiovascular diseases in Saudi Arabia. BMC Cardiovasc Disord.

[4] Alhabib KF, Batais MA, Almigbal TH, Alshamiri MQ, Altaradi H, Rangarajan S, Yusuf S (2020). Demographic, behavioral, and cardiovascular disease risk factors in the Saudi population: results from the Prospective Urban Rural Epidemiology study (PURE-Saudi). BMC Public Health.

[5] Mulle JG, Vaccarino V (2013). Cardiovascular disease, psychosocial factors, and genetics: the case of depression. Prog Cardiovasc Dis.

[6] Clark AM, DesMeules M, Luo W, Duncan AS, Wielgosz A (2009). Socioeconomic status and cardiovascular disease: risks and implications for care. Nat Rev Cardiol.

[7] Albus C (2010). Psychological and social factors in coronary heart disease. Ann Med.

[8] Zhang M, Shi Y, Yang Y, Liu L, Xiao J, Guo T, Li J (2017). Burnout is associated with poor recovery of physical performance and low quality of life in patients after their first episode of acute coronary syndrome: a hospital-based prospective cohort study. Int J Cardiol.

[9] Melamed S, Shirom A, Toker S, Berliner S, Shapira I (2006). Burnout and risk of cardiovascular disease: evidence, possible causal paths, and promising research directions. Psychol Bull.

[10] Franza F (2019). The emotional and psychological burden of the "burnout" in families of psychiatric patients. Psychiatr Danub.

[11] Demerouti E, Bakker AB, Vardakou I, Kantas A (2003). The convergent validity of two burnout instruments: a multitrait-multimethod analysis. Eur J Psychol Assess.

[12] Rosenman RH, Brand RJ, Sholtz RI, Friedman M (1976). Multivariate prediction of coronary heart disease during 8.5 year follow-up in the Western Collaborative Group Study. Am J Cardiol.

[13] Orenstein GA, Lewis L (2022). Erikson's Stages of Psychosocial Development. https://www.ncbi.nlm.nih.gov/books/NBK556096/.

[14] Knight ZG (2017). A proposed model of psychodynamic psychotherapy linked to Erik Erikson's eight stages of psychosocial development. Clin Psychol Psychother.

[15] Branje S, Koper N (2018). Psychosocial development. The SAGE Encyclopedia of Lifespan Human Development.

[16] Samkry A (2021). The relationship between positive thinking and ego virtues among coronavirus pandemic in Saudi Arabia. Educ J Fac Educ Sohag.

[17] Markstrom CA, Sabino VM, Turner BJ, Berman RC (1997). The psychosocial inventory of ego strengths: development and validation of a new Eriksonian measure. J Youth Adolesc.

[18] (2022). Sample size calculator. http://www.raosoft.com/samplesize.html.

[19] Leclercq C, Braeckman L, Firket P, Babic A, Hansez I (2021). Interest of a joint use of two diagnostic tools of burnout: comparison between the Oldenburg burnout inventory and the early detection tool of burnout completed by physicians. Int J Environ Res Public Health.

[20] Alghamdi HA (2010). Ego Effectiveness Scale.

[21] Tash AA, Al-Bawardy RF (2023). Cardiovascular disease in Saudi Arabia: facts and the way forward. J Saudi Heart Assoc.

[22] (2022). Cardiovascular disease: a public health priority. https://www.shc.gov.sa/Arabic/NHC/Activities/Documents/Cardiovascular%20disease%20in%20KSA%20-%20a%20public%20health%20priority.pdf.

[23] Nagarajan R, Ramachandran P, Dilipkumar R, Kaur P (2024). Global estimate of burnout among the public health workforce: a systematic review and meta-analysis. Hum Resour Health.

[24] Alhafithi M, Al-Dubai SA, Alalwani SS, Masarit AM (2022). Prevalence and associated factors of burnout among nurses in a general hospital in Yanbu, Saudi Arabia. Middle East J Fam Med.

[25] Al Owa KR, Valentine A, Bakare A, Akanmu IY, Adeboye A (2021). Prevalence and factors of burnout among healthcare workers in Eastern Province, Saudi Arabia. Open J Soc Sci.

[26] Qedair JT, Balubaid R, Almadani R, Ezzi S, Qumosani T, Zahid R, Alfayea T (2022). Prevalence and factors associated with burnout among nurses in Jeddah: a single-institution cross-sectional study. BMC Nurs.

[27] Karim Alenezi N, Hamad Alyami A, Omar Alrehaili B, Adnan Arruhaily A, Kareem Alenazi N, Abdo Radman Al-Dubai S (2022). Prevalence and associated factors of burnout among Saudi resident doctors: a multicenter cross-sectional study. Alpha Psychiatry.

[28] Bu Bshait MS, Al Abdulqader AA, Almaqhawi AK (2024). Burnout among emergency and surgery residents: an exploration of contributing factors and implications. Saudi Med J.

[29] Garg PK, Claxton JS, Soliman EZ, Chen LY, Lewis TT, Mosley T, Alonso A (2021). Associations of anger, vital exhaustion, anti-depressant use, and poor social ties with incident atrial fibrillation: the Atherosclerosis Risk in Communities Study. Eur J Prev Cardiol.

[30] John A, Bouillon-Minois JB, Bagheri R (2024). The influence of burnout on cardiovascular disease: a systematic review and meta-analysis. Front Psychiatry.

[31] Kivimäki M, Nyberg ST, Batty GD (2017). Long working hours as a risk factor for atrial fibrillation: a multi-cohort study. Eur Heart J.

[32] Chen Y, Juvinao-Quintero D, Velez JC, Muñoz S, Castillo J, Gelaye B (2023). Personal and work-related burnout is associated with elevated diastolic blood pressure and diastolic hypertension among working adults in Chile. Int J Environ Res Public Health.

[33] Singh C, Singh G (2022). Measuring the impact of ego strength among type 2 diabetes. J Posit Sch Psychol.

[34] Sirohi S, Monika Monika (2017). Study of ego strength in relation to stress management among senior secondary students. Int Educ Res J.

[35] Sankhyan P (2022). Ego strength and self actualization in relation to burn out among teachers working in Government Primary Schools, District Bilaspur, Himachal Pradesh. Int J Creat Res Thoughts.

[36] Kaur D, Javed N (2023). Ego strength and burnout among female teachers. Indian Educ Res J.

[37] (2023). Medscape national physician burnout, depression & suicide report 2019. Report.

[38] Yan L, Zhong X, Yang L, Long H, Ji P, Jin X, Liu L (2022). Gender differences in job burnout, career choice regret, and depressive symptoms among Chinese dental postgraduates: a cross-sectional study. Front Public Health.

[39] Markus C, Daniela B, Paul J (2018). Gender differences in different dimensions of common burnout symptoms in a group of clinical burnout patients. Neuropsychiatry.

[40] Artz B, Kaya I, Kaya O (2022). Gender role perspectives and job burnout. Rev Econ Househ.

[41] Redondo-Flórez L, Tornero-Aguilera JF, Ramos-Campo DJ, Clemente-Suárez VJ (2020). Gender differences in stress- and burnout-related factors of university professors. Biomed Res Int.

[42] Alenezi L, Gillespie GL, Smith C, Davis KG (2024). Gender differences in burnout among US nurse leaders during COVID-19 pandemic: an online cross-sectional survey study. BMJ Open.

[43] Wang J, Zhang L, Jiang F, Liu Y, Wang M, Wu Y, Tang YL (2022). Gender differences in burnout among endocrinologists in China. Front Psychol.

[44] Treichler EB, Palmer BW, Wu TC (2021). Women and men differ in relative strengths in wisdom profiles: a study of 659 adults across the lifespan. Front Psychol.

[45] Levit DB (1991). Gender differences in ego defenses in adolescence: sex roles as one way to understand the differences. J Pers Soc Psychol.

[46] Alagh MT, Ghosh S (2022). Impact of ego virtues in middle and old ages: a comparative study. Int J Indian Psychol.

